# Oral Nutritional Supplement Adherence and Nutritional Outcomes in Hemodialysis Patients—A Prospective Study

**DOI:** 10.3390/jcm14238337

**Published:** 2025-11-24

**Authors:** Lea Katalinic, Ivana Juric, Armin Atic, Bojan Jelakovic, Nikolina Basic-Jukic

**Affiliations:** Department of Nephrology, Arterial Hypertension, Dialysis and Transplantation, University Hospital Centre Zagreb, 10000 Zagreb, Croatia

**Keywords:** hemodialysis, oral nutritional supplements, adherence, protein-energy wasting, malnutrition

## Abstract

**Background/Objectives**: Protein-energy wasting (PEW) affects up to 75% of hemodialysis (HD) patients, yet adherence to oral nutritional supplements (ONSs) remains poorly understood. This study evaluated ONS adherence patterns, associated socio-demographic and psychological factors, and clinical outcomes over 24 months in chronic HD patients. **Methods**: A 24-month prospective study was conducted in 101 HD patients. Adherence was assessed using the 4-item Morisky Medication Adherence Scale (MMAS-4), and depressive symptoms with the Beck Depression Inventory (BDI). Nutritional status was evaluated using the Malnutrition–Inflammation Score (MIS) and anthropometric measurements. Laboratory markers were obtained. Individualized nutritional education was provided at each visit. **Results**: Regular ONS use was reported in 50.5% of patients. High adherence (MMAS-4 = 0) was observed in 36.6% of the cohort. Forgetfulness (45.3%) and adverse effects (34.4%) were the most common obstacles. Adherence was significantly associated with sex (*p* = 0.007), with men more frequently demonstrating low adherence. Education level showed a weak, but significant positive correlation with MMAS-4 score (Spearman’s ρ = 0.25, *p* = 0.018), indicating slightly lower adherence among more educated patients. MMAS-4 and BDI scores were positively correlated (Spearman’s ρ = 0.25, *p* = 0.04), indicating that greater depressive symptom burden was associated with lower adherence. Regular ONS users demonstrated improved nutritional status (lower MIS; 9 vs. 7, *p* < 0.001), higher hemoglobin (106 vs. 114 g/L, *p* = 0.03), and increased mid-upper arm circumference (MUAC; 26 vs. 28 cm, *p* = 0.02). Lean tissue mass was preserved over time (*p* = 0.009). However, individualized education had limited effect on patients with initially low adherence. Individualized nutritional education was associated with improved acceptance and implementation of recommendations. Over two years of follow-up, nutritional education was associated with preserved lean and fat tissue index (LTI, *p* = 0.009; FTI, *p* = 0.08), reductions in interdialytic weight gain, and significant improvements in MUAC, waist circumference, and scapular skinfold thickness (*p* = 0.03; *p* < 0.001; *p* = 0.02). Prealbumin and hemoglobin levels also increased significantly (*p* = 0.02; *p* = 0.04). However, education alone was insufficient for certain subgroups, particularly older patients and those initially classified as non-adherent. During follow-up, 17 patients died. Lower MUAC (OR = 2.97, 95% CI: 1.45–6.08) and triceps skinfold thickness (OR = 1.37, 95% CI: 1.12–1.68) were the strongest independent predictors of mortality. **Conclusions**: Adherence to ONSs remains suboptimal in HD patients. Individualized nutritional education was associated with improved adherence and nutritional status in some subgroups but may be insufficient in older or initially non-adherent patients. Simple anthropometric markers are strong mortality predictors and may offer practical value for routine monitoring.

## 1. Introduction

Nutritional disorders are recognized as one of the late complications of chronic kidney disease (CKD). The interplay of malnutrition, chronic inflammation, and comorbidities—further aggravated by dialysis-specific factors—contributes accelerated atherosclerosis and cardiovascular disease. This process, known as the Malnutrition–Inflammation–Atherosclerosis (MIA) syndrome, is recognized as a significant predictor of mortality [1,2].

Signs of malnutrition have been reported in 18–75% of dialysis patients, with considerable variability largely related to inconsistent diagnostic criteria and terminology [3]. To improve diagnostic precision, the term protein-energy wasting (PEW) was introduced into clinical practice in 2007 [4,5].

PEW is defined by insufficient protein and energy intake accompanied by characteristic laboratory and anthropometric changes, most notably progressive loss of muscle mass and visceral fat [5]. The driving factor behind the complex pathophysiological mechanisms leading to this is the inability to maintain adequate nutrient intake due to anorexia or numerous dietary restrictions [6,7]. Therefore, individualized nutritional counseling and ongoing monitoring should be central components of multidisciplinary dialysis care [4,8].

When protein and energy intake becomes insufficient, oral nutritional supplements (ONSs) may be a practical and effective first-line intervention for preventing or treating PEW [9,10]. However, real-world adherence to prescribed ONSs is insufficiently understood, and non-adherence may reduce the effectiveness of nutritional interventions.

The aim of this prospective study was to examine the prevalence and determinants of ONS adherence in chronic HD patients, and to evaluate the impact of individualized nutritional education and ONS use on nutritional and clinical outcomes over 24 months.

## 2. Materials and Methods

We conducted a 24-month prospective study at the Dialysis Unit, University Hospital Centre (UHC) Zagreb, in patients undergoing maintenance hemodialysis (HD). The study was approved by the Medical Ethics Committee (protocol code 8.1.-14/121-2, number: 02/21/JG; approval date: 25 February 2015). Exclusion criteria were active infection, autoimmune disease, untreated or disseminated malignancy, and expected survival <24 months.

Demographic and clinical data—including age, sex, education level, marital and household status, comorbidities, primary kidney disease, and HD vintage—were obtained from medical records.

The primary objective of this study was to determine the prevalence of non-adherence to prescribed ONSs in patients undergoing chronic HD and to assess the impact of individualized nutritional education on adherence over 24 months.

The secondary objectives were to

(1) Identify socio-demographic, psychological, and clinical factors associated with ONS adherence;
(2) Evaluate the effects of adherence on nutritional status, body composition, and laboratory markers; and
(3) Examine the relationships between adherence, nutritional status, and mortality.

Outcomes were assessed using the 4-item Morisky Medication Adherence Scale (MMAS-4) for adherence, the Malnutrition–Inflammation Score (MIS), anthropometric measurements, bioimpedance-derived body composition, laboratory analyses, and the 21-item Beck Depression Inventory (BDI) scores. Mortality and adverse events were recorded throughout the study period.

Medication adherence was assessed using the MMAS-4, where 0 indicates high adherence, 1–2 moderate adherence, and 3–4 low adherence [11]. Depressive symptoms were assessed using the BDI, and categorized as minimal/normal (0–10), mild (11–16), borderline (17–20), moderate (21–29), or severe (≥30) [12].

Blood samples were collected before a mid-week HD session for routine laboratory testing, including complete blood count, creatinine, urea, electrolytes, total protein, albumin, prealbumin, iron studies (serum iron, total iron-binding capacity (TIBC), ferritin), lipid profile, and C-reactive protein (CRP).

Anthropometric measurements included neck circumference (NC), mid-upper arm circumference (MUAC), waist circumference (WC), and hip circumference (HC). Skinfold thickness was measured at the triceps (TST) and scapular (SST) regions using a caliper. Dry body weight (BW) and height were used to calculate body mass index (BMI). Body composition (lean tissue index—LTI, fat tissue index—FTI, overhydration—OH) was assessed using bioimpedance spectroscopy (Fresenius Medical Care Body Composition Monitor, BCM). Nutritional status was further evaluated with the MIS, a validated tool for PEW [13].

Individualized nutritional education and ONS prescription was delivered using illustrative materials and sample meal plans at baseline, 12 months, and 24 months.

### Statistical Analysis

Analyses were performed using Stata/SE 11.2 (StataCorp LLC., College Station, TX, USA). Data distribution was assessed with the Shapiro–Wilk test, and homogeneity of variance with the F-test. Normally distributed variables are presented as mean ± SD, while non-normally distributed variables are expressed as median (range). Categorical variables are reported as frequencies and percentages.

Adherence was analyzed both as a continuous MMAS-4 score and as categorical adherence levels (high: 0; moderate: 1–2; low: 3–4). Group differences were assessed using Student’s *t*-test or Mann–Whitney U test for two-group comparisons, and one-way ANOVA or Kruskal–Wallis tests for comparisons across more than two groups, as appropriate. Associations between categorical variables were evaluated using Pearson’s χ^2^ test and effect size was expressed using Cramer’s V.

Correlations between continuous variables were assessed using Spearman’s rank correlation coefficients. Longitudinal changes in nutritional and body composition parameters were analyzed in the survivors-only cohort (patients who completed all three study visits) to minimize survival bias.

To identify predictors of mortality, multivariable stepwise logistic regression was performed, including variables with *p* < 0.10 in univariate analyses while avoiding collinearity between related parameters.

## 3. Results

### 3.1. Cohort Characteristics

A total of 101 patients undergoing maintenance HD were enrolled: 57 males (56.4%) and 44 females (43.6%), with a mean age of 60.8 ± 16.2 years. The most common causes of end-stage kidney disease (ESRD) were glomerulonephritis (27.7%), diabetic nephropathy (21.8%), and hypertensive nephropathy (14.9%). The median HD vintage was 51 months (range 9–456), and all patients received bicarbonate HD for at least 3 h per session, 2–4 times per week, using high-flux polysulfone dialyzers and ultrapure dialysate (flow rate 500 mL/min).

Most patients (75.2%) completed secondary or higher education (52.5% secondary, 18.8% higher), while 26.7% had primary education only. Fifty-seven patients (56.4%) were married, 41 (40.6%) were single, divorced, or widowed, and 3 (3.0%) did not report marital status. There were no significant differences between men and women in either education level or marital status (*p* > 0.05).

The mean baseline MIS was 7.8 ± 3.4, consistent with mild to moderate malnutrition risk. Patients with lower MIS had significantly higher BMI (27.68 ± 4.21 vs. 23.32 ± 3.89 kg/m^2^, *p* = 0.004), whereas serum albumin levels did not differ significantly between groups (38.77 ± 4.12 vs. 37.11± 5.23 g/L, *p* = 0.33).

Baseline cohort characteristics are summarized in Table 1.

### 3.2. Adherence and Mood

The mean MMAS-4 score in the cohort was 1.5 ± 1.2 (range 0–4). Based on MMAS-4 categories, 37 patients (36.6%) demonstrated high adherence, 45 (44.6%) moderate adherence, and 19 (18.8%) low adherence. The distribution of adherence categories did not differ significantly across age groups (*p* = 0.71).

Adherence was significantly associated with sex (χ^2^(2) = 9.93, *p* = 0.007), with women more frequently demonstrating moderate adherence, while men were more often among those with low adherence. Marital status and living situation did not significantly influence adherence (*p* = 0.16 and *p* = 0.21, respectively).

Although adherence patterns varied by educational level, the categorical comparison did not reach statistical significance (χ^2^, *p* = 0.19). However, when analyzed as continuous variables, MMAS-4 scores demonstrated a weak but statistically significant positive correlation with higher education level (Spearman’s ρ = 0.25, *p* = 0.018), indicating that patients with higher education tended to have slightly lower adherence. The effect size of this association was small (Cramér’s V = 0.19). These associations are summarized in Table 2.

The most common reasons for non-adherence were forgetfulness (45.3%), adverse effects (34.4%), being away from home (17.2%), and the perception of feeling well without treatment (12.5%).

**Figure 1:**
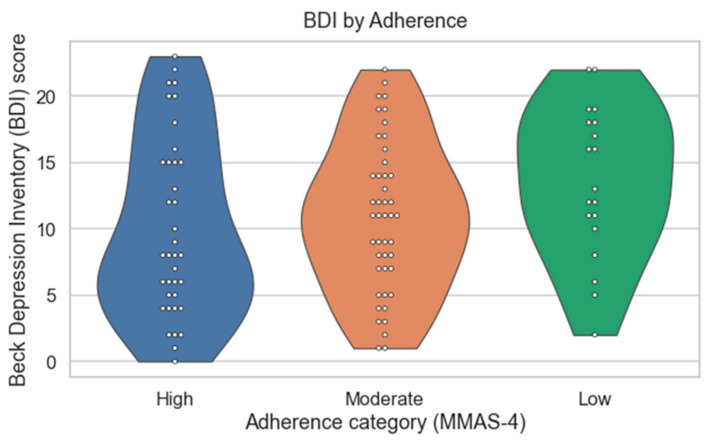
Distribution of Beck Depression Inventory (BDI) scores across adherence categories (MMAS-4). Violin plots show the distribution of BDI scores within each adherence category (MMAS-4). When analyzed as a continuous variable, MMAS-4 score demonstrated a positive correlation with BDI score (Spearman’s r_s_ = 0.25, *p* = 0.04), indicating that higher depressive symptom burden was associated with lower adherence.

The mean BDI score was 11.2 ± 6.8 (range 0–23), with most participants showing minimal or mild depressive symptoms and no cases of severe depression. BDI scores tended to be higher among patients with lower adherence, although this difference was not statistically significant when adherence categories were compared directly (*p* = 0.19). When analyzed as continuous variables, however, MMAS-4 and BDI scores were positively correlated (Spearman’s ρ = 0.25, *p* = 0.04), indicating that greater depressive symptom burden was associated with lower adherence. This relationship is illustrated in Figure 1.

### 3.3. Adherence to Oral Nutritional Supplement (ONS) Use and Acceptance of Nutritional Recommendations

ONS was used by 50.5% of patients (*n* = 51). Duration of consumption varied: 35 patients (34.7%) used ONS for ≥3 months, 7 patients (6.9%) for 6–12 months, and 6 patients (5.9%) for >12 months. Regarding daily consumption, 45 patients (44.6%) consumed one bottle daily, while 11 patients (10.9%) consumed two or more bottles daily. Among non-users, reasons included perceiving ONS as unimportant (14.9%), not being prescribed (5%), inability to take it (3%), or intolerance/nausea (4%).

At the first two visits, ONS users were significantly more likely to have high MISs (*p* = 0.02, and *p* = 0.01), indicating that supplementation was more frequently prescribed in patients with worse baseline nutritional status. By the third visit, this difference was no longer significant (*p* = 0.29), suggesting a convergence of nutritional status over time between ONS users and non-users (Table 3).

By the end of the study, regular ONS users showed improvements in several nutritional markers: MIS decreased from median 9 (IQR: 6–10) to 7 (IQR: 5–9), *p* < 0.001. Hemoglobin levels increased from 106 g/L (IQR: 97–114) to 114 g/L (IQR: 101–120), *p* = 0.03. MUAC increased from 26 cm (IQR: 24–28) to 28 cm (IQR: 25–30), *p* = 0.02), and WC from 88 cm (IQR: 79–100) to 95.5 cm (IQR: 84.1–103.3), *p* = 0.04. Body composition analysis showed preservation of lean tissue mass in both LTI and FTI, corresponding with reduced overhydration from 2.4 L (IQR: 1.5–3.4) to 1.8 L (IQR: 0.7–3.0), *p* = 0.02.

Patients who consumed ONS for 6–12 months showed higher pulse pressure compared with the other groups (ANOVA: F = 4.872, *p* = 0.015). More frequent ONS intake was also associated with higher systolic blood pressure values (169.4 ± 40.7 mmHg vs. 148.3 ± 21.5 mmHg for single-bottle users, *p* = 0.02), as well as higher serum calcium levels (F = 3.769, *p* = 0.036) and HDL cholesterol (F = 3.946, *p* = 0.031).

Overall, patients who continued to take ONS over time tended to maintain or slightly improve their nutritional status, while those who did not use ONS showed more variable patterns. Because patients with poorer nutritional status and lower adherence were also more likely to die during follow-up, the changes across visits may partly reflect survival bias. To address this, a longitudinal analysis was performed; these results are presented in Section 3.4.

### 3.4. Longitudinal Changes

One year after individualized nutritional counseling, 74 patients (73.3%) reported actively following the recommendations, while 27 patients (26.7%) did not. The most common reasons for non-adherence were the perception that recommendations were not important (*n* = 15), lack of clarity or recall regarding the recommendations (*n* = 5), inadequate conditions to implement dietary changes (*n* = 4), or adverse effects leading to discontinuation (*n* = 3).

Patients with medium or higher education were more likely to accept nutritional recommendations (*p* = 0.08), whereas those with lower education showed no difference between acceptance and non-acceptance. Larger household size (≥3 members) was also associated with greater likelihood of acceptance (*p* = 0.08). Age, sex, and marital status were not associated with recommendation acceptance (*p* > 0.05).

Over the 24-month follow-up period, with repeated nutritional education, patients in the overall cohort demonstrated preserved lean tissue mass (LTI, *p* = 0.009), with stable fat tissue index (FTI, *p* = 0.08). Interdialytic weight gain decreased (*p* = 0.01), and MUAC, WC, and SST improved (*p* = 0.03; *p* < 0.001; *p* = 0.02). Prealbumin increased (*p* = 0.02), and hemoglobin rose modestly (*p* = 0.04) independent of iron or erythropoietin (EPO) supplementation. Among other biochemical parameters, fasting glucose and potassium improved significantly (*p* = 0.02; *p* < 0.001), while the remaining parameters remained stable. These results for all three study visits are summarized in Table 4. Because patients with poorer nutritional status and lower adherence were more likely to die during follow-up, a survivor-only longitudinal analysis was performed as a sensitivity analysis to address potential survival bias. This analysis demonstrated a similar pattern of nutritional stabilization with modest improvement over time, rather than the progressive decline typically expected in chronic hemodialysis patients. The survivors-only longitudinal analysis has been added to the Supplementary Materials (Table S1).

Continuous nutritional education contributed to improved adherence in a subset of patients, particularly those younger than 65 years and those who had moderate adherence at baseline. After targeted education, patients with medium or higher education demonstrated significant improvement in adherence over time (*p* = 0.004), while patients with lower education generally maintained high adherence from baseline. Patients classified as non-adherent at baseline did not show meaningful improvement despite repeated interventions. Marital status, living situation, and household size did not influence changes in adherence over time (*p* > 0.05). BDI scores were not associated with nutritional outcomes or response to nutritional intervention (*p* > 0.05). However, the most adherent patients consistently exhibited the lowest depressive symptom levels, although this difference did not reach statistical significance.

### 3.5. Mortality Predictors

Seventeen patients died during the study period, with all deaths occurring during the first two study visits and none at the final assessment. Mortality was highest among older age groups (66–75 years: 41%; ≥76 years: 35%; *p* = 0.06), and deceased patients were significantly older than survivors (71.58 vs. 58.64 years, *p* = 0.002). There were no significant differences in sex, education level, or marital status. Diabetic nephropathy was the most frequent primary kidney disease among deceased patients (29%, *p* = 0.001), and cerebrovascular events represented the leading cause of death (35.3%).

Deceased patients exhibited significantly poorer nutritional profile, characterized by higher MIS (10.2 ± 3.1 vs. 7.8 ± 3.3, *p* = 0.008), lower LTI (10.07 ± 2.45 vs. 13.00 ± 3.21 kg/m^2^, *p* = 0.0001), and lower creatinine (678.1 ± 156.3 vs. 789.9 ± 198.7 µmol/L, *p* = 0.033) and albumin (35.2 ± 4.8 vs. 38.9 ± 4.2 g/L, *p* = 0.01) levels. Anthropometric measures similarly reflected poorer nutritional reserves, with lower TST (14.24 ± 4.12 vs. 16.51 ± 5.23 mm, *p* < 0.001), and lower MUAC (25.1 ± 3.2 vs. 27.5 ± 3.8 cm, *p* = 0.02) in non-survivors. Psychosocial parameters also differed, with lower adherence (MMAS-4: 2.4 ± 1.3 vs. 1.3 ± 1.1, *p* = 0.006) and higher depressive symptom burden (BDI: 14.8 ± 6.2 vs. 10.5 ± 6.7, *p* = 0.007) observed among deceased patients.

Multivariate logistic regression identified MUAC and TST as independent predictors of mortality. The final model was statistically significant (χ^2^ = 35.1, df = 5, *p* < 0.001), explained 38.2–69.4% of the variance (Nagelkerke R^2^), and correctly classified 93.2% of cases. MUAC (OR = 2.97, 95% CI: 1.36–6.49, *p* = 0.006) and TST (OR = 1.37, 95% CI: 1.09–1.73, *p* = 0.008) were the strongest predictors of adverse outcomes. The full regression model is provided in the Supplementary Material (Table S2).

## 4. Discussion

With a prevalence between 5% and 15%, CKD is reaching epidemic proportions and has become a major global public health challenge [14]. With the increasing prevalence of CKD, the proportion requiring initiation of dialysis therapy is also steadily increasing [15]. Despite advances in dialysis techniques and traditional risk factor management, mortality in this population remains inexplicably high. In recent years, nutritional status has been recognized as one of the most important non-traditional prognostic factors in HD patients, with PEW and inflammation strongly linked to adverse outcomes [15,16].

Dietary management in HD is among the most complex in clinical practice, which makes adherence particularly challenging. Previous studies suggest that adherence to dietary recommendations is multifactorial—mainly related to insufficient knowledge, as well as psychosocial and sociodemographic determinants [17,18]. Adherence rates vary widely, with non-adherence reported in up to 50% of HD patients, underscoring the need to better understand these barriers [19,20].

In our study, only half of the cohort regularly consumed ONS. Even among those with prescriptions, compliance varied. Forgetfulness, adverse effects, and lack of perceived importance were the most frequently reported obstacles. These findings are consistent with previous research showing that non-adherence to nutritional interventions is multifactorial—often influenced by patient perceptions, taste intolerance, and disruptions to daily routines [21,22].

Patients who regularly used ONS showed favorable changes in nutritional parameters, including reductions in MIS, higher hemoglobin levels, and maintenance of lean mass. ONS consumption over the longer periods (6–12 months) was associated with higher pulse and systolic blood pressure, as well as improved calcium and HDL levels, suggesting a complex interaction between nutritional intake, cardiovascular parameters, and metabolic status. This highlights the potential clinical benefits of ONS when adherence is achieved, supported by several other observational studies that reported improved albumin levels and anthropometric measures among dialysis patients receiving ONS [23,24,25,26]. A meta-analysis by Liu et al. further confirmed that ONS use can improve nutritional status and muscle function without worsening electrolyte disturbances [27]. However, the fact that many patients failed to achieve these benefits emphasizes the need for individualized strategies to improve adherence.

Individualized nutritional education was linked to better acceptance and implementation of dietary recommendations in our cohort. Over two years of follow up, repeated education was associated with improvements in MUAC, WC, and albumin levels, as well as preserved lean tissue mass (LTI and FTI). These findings are consistent with previous studies emphasizing that structured and repeated educational interventions can improve nutritional outcomes in HD patients [28,29]. However, education alone was insufficient for certain subgroups, particularly those initially non-adherent, indicating the importance of tailored educational strategies.

In terms of adherence, our study found a significant association with sex, where women more frequently demonstrated better adherence, while men were more often represented in the low-adherence group. Age, marital status and living situation did not influence adherence. Although education level did not show significant differences in adherence categories, continuous variable analysis demonstrated a weak but statistically significant positive correlation between higher educational level and higher MMAS-4, suggesting slightly lower adherence among highly educated patients. Interestingly, patients with lower educational levels were more likely to achieve perfect compliance [30]. Although earlier studies identified low education as a risk factor for non-adherence [31,32], newer research reported no clear relationship [33,34]. It seems that perceived relevance of therapy, trust in medical recommendations, and clarity of treatment goals play a greater role in shaping adherence than formal educational level [35].

Our findings further highlight the important association between mood and adherence. Depressive symptoms were present in approximately one-quarter of the cohort, which is consistent with previous reports estimating the prevalence of depression in 20–40% of patients undergoing HD [36]. The clinical overlap between uremic and depressive symptoms—such as fatigue, sleep disturbances, and appetite changes—complicates detection, but depression remains an important factor influencing patient outcomes [37,38]. In our study, patients aged 56–65 years and those living alone were most likely to report depressive symptoms, while patients with higher educational level were least affected. Notably, sex did not significantly influence depression rates, in contrast to some earlier studies suggesting female sex as a risk factor [39]. Importantly, patients with lower BDI scores were also those with the highest adherence, supporting evidence that depressive symptoms negatively affect treatment engagement, including medication adherence, dialysis attendance, fluid restriction, and dietary compliance. These observations are consistent with prior studies documenting both elevated rates of depressive symptoms in dialysis populations and their association with poorer adherence and clinical outcomes [40,41,42,43]. The most frequently reported barriers to ONS adherence in our study—forgetfulness, adverse effects, being away from home, and the perception of not needing supplementation—mirror findings from previous research and underscore the multifactorial nature of adherence challenges in this patient group [32,34,35,44].

Individualized nutritional education was associated with better implementation of dietary recommendations in the majority of patients. This is consistent with previous nutritional education trials demonstrating that structured and continuous education is associated with significant improvements in nutritional status [45,46,47,48,49,50]. For example, a randomized controlled trial by Dsouza et al. showed that structured education significantly improved adherence to dietary and fluid restrictions, dialysis attendance, and medication regimens [45]. Findings regarding the influence of social support on adherence have been mixed [51,52]. In a systematic review by Sousa et al., approximately 44% of studies reported a positive association between social support and adherence to dietary restrictions [53], and Alatawi et al. similarly found that higher perceived support from family or close contacts correlated with better adherence across dietary, fluid, and medication regimens [54]. Nonetheless, patients who were older or initially non-adherent showed limited response to education alone, suggesting that conventional educational strategies may be insufficient for certain subgroups.

Mortality was the highest among older patients and those with diabetic nephropathy. Deceased patients had poorer nutritional status and lower adherence at baseline. MUAC and TST emerged as the strongest independent predictors of mortality, underscoring the clinical importance of simple anthropometric markers in routine nutritional evaluation. These findings support prior research emphasizing the prognostic relevance of preserved lean mass in HD [5,55,56,57].

This study has several limitations, including the relatively small sample size and the single-center design. Differences in group sizes, particularly between surviving and deceased patients, may have limited statistical power for some comparisons. In addition, adherence was assessed using a self-reported scale, which may overestimate actual compliance. Despite these limitations, the longitudinal design and the integration of clinical, biochemical, anthropometric, and psychosocial measures provide a comprehensive and clinically relevant perspective on the factors influencing ONS adherence and clinical outcomes in HD patients.

Future studies should evaluate modern and structured multidisciplinary approaches to support long-term adherence. Addressing depressive symptoms may have additional indirect benefits by improving both nutritional status and treatment engagement.

Overall, our findings indicate that adherence to ONS—and treatment in general—in HD patients is shaped by a complex interplay of nutritional, psychological, and social factors. Patient-centered strategies that combine individualized education, psychosocial support, and ongoing monitoring may represent the most effective path to improving adherence and long-term clinical outcomes.

## 5. Conclusions

Prescribing ONSs alone is not sufficient to maintain adequate nutritional status in patients undergoing HD. Improving adherence requires a multi-component approach that combines repeated and personalized education, attention to patient perceptions and palatability, and appropriate social and family support—particularly for older patients and those with lower baseline adherence. Future research should focus on integrated, multidisciplinary interventions tested in randomized designs, with longer follow-up and objective measures of adherence, to identify which strategies are most effective in sustaining nutritional improvements in this vulnerable population.

## Figures and Tables

**Table 1:** Baseline patients’ characteristics.

Characteristics	All Patients (*n* = 101)
**Demographic**	
Gender M/F (%)	56.4/43.6%
Age (mean, std; years)	60.8 ± 16.15
HD vintage (median; months)	51 (range 9–456)
**Primary kidney disease (%)**	
Glomerulonephritis	27.7%
Diabetic nephropathy	21.8%
Hypertensive kidney disease	14.9%
Other	35.6%
**Sociodemographic and psychosocial determinants**	
**Educational level (%)**	
Primary education	26.7%
Secondary education	52.5%
Higher education	18.8%
**Marital status (%)**	
Married	56.4%
Single/divorced/widowed	40.6%
Did not specify	3.0%
**MMAS-4** (mean, std)	1.5 ± 1.2
**Categories (%):**	
Low adherence	18.8%
Moderate adherence	44.6%
High adherence	36.6%
**BDI** (mean, std)	11.2 ± 6.8
**Categories (%):**	
Minimal/normal mood oscillation	46.5%
Mild depression	31.7%
Borderline depression	14.9%
Moderate depression	6.9%
Severe depression	0%
**Nutritional parameters (mean, std)**	
MIS	7.8 ± 3.4
BMI (kg/m^2^)	25.2 ± 5.4
BW (kg)	69.8 ± 17.2
NC (cm)	38.3 ± 4.3
MUAC (cm)	27.6 ± 4.5
WC (cm)	94.3 ± 14.8
HC (cm)	100.7 ± 10.9
TST (mm)	15.5 ± 7.7
SST (mm)	15.4 ± 7.4
LTI (kg/m^2^)	12.5 ± 3.1
FTI (kg/m^2^)	12.3 ± 5.3
OH (L)	2.6 ± 1.6
**Laboratory parameters (mean, std)**	
Hemoglobin (g/L)	107.6 ± 12.2
Creatinine (µmol/L)	771.1 ± 198.3
Urea (mmol/L)	20.9 ± 5.5
Albumin (g/L)	37.8 ± 3.3
Prealbumin (g/L)	0.5 ± 0.2
Calcium (mmol/L)	2.2 ± 0.2
Phosphorus (mmol/L)	1.4 ± 0.4
Potassium (mmol/L)	4.9 ± 0.6
Cholesterol (mmol/L)	4.2 ± 1.1
Triglycerides (mmol/L)	1.7 ± 0.9
Glucose (mmol/L)	6.5 ± 2.7
Iron (µmol/L)	11.8 ± 4.8
TIBC	40.3 ± 8.2
Ferritin (µg/L)	388.3 ± 189.2
CRP (mg/L)	8.2 ± 12.7

**Table 2:** Association between adherence (MMAS-4 categories) and socio-demographic and clinical parameters; *p* < 0.05 is considered statistically significant.

Parameter	High Adherence (MMAS-4 = 0)	Moderate Adherence (MMAS-4 = 1–2)	Low Adherence (MMAS-4 = 3–4)	*p*-Value	Test
**Age group, *n* (%)**				**0.71**	χ^2^
≤55 years	10	11	5		
56–65 years	9	14	8		
66–75 years	10	13	2		
≥75 years	8	7	4		
**Sex (F/M), *n***	13/24	27/18	4/15	**0.007**	χ^2^
**Marital status, *n* (%)**				**0.16**	χ^2^
Married/partnered	18	29	10		
Single/widowed/divorced	19	13	9		
**Living situation, *n* (%)**				**0.21**	χ^2^
Living alone	14	12	3		
Living with others	23	33	16		
**Education level, *n* (%)**				**0.19**	χ^2^
Primary	15	10	2		
Secondary	17	22	14		
Higher	2	3	2		
**BDI category, *n* (%)**				**0.19**	χ^2^
Minimal (0–10)	22	20	5		
Mild (11–16)	8	16	7		
Borderline (17–20)	3	7	5		
Moderate (21–29)	4	2	2		
Severe (≥30)	0	0	0		
**MIS, median (IQR)**	8 (5–10)	8 (5–10.8)	8.5 (6.3–10.5)	**0.46**	Kruskal–Wallis
**Albumin (g/L)**	39 (37–40)	38 (36–40)	37 (33–40)	**0.41**	ANOVA
**LDL (mmol/L)**	2.1 (1.7–2.4)	2.5 (2.0–3.0)	1.8 (1.2–2.6)	**0.038**	ANOVA

**Table 3:** Number of participants (%) stratified by degree of inflammation and ONS intake. Statistically significant difference in the observed parameters (Chi-square test *p* < 0.05).

	Proportion of Participants by ONS Intake (N)			*p* *
	ONS Non-Users	ONS Users	Total	
**MIS first visit**				
Low and moderate inflammation	26 (59)	18 (35)	44 (46)	**0.02**
High inflammation	18 (41)	33 (65)	51 (54)	
Total	44 (100)	51 (100)	95 (100)	
**MIS second visit**				
Low and moderate inflammation	21 (62)	18 (34)	39 (45)	**0.01**
High inflammation	13 (38)	35 (66)	48 (55)	
Total	34 (100)	53 (100)	87 (100)	
**MIS third visit**				
Low and moderate inflammation	19 (73)	26 (60)	45 (65)	0.29
High inflammation	7 (27)	17 (40)	24 (35)	
Total	26 (100)	43 (100)	69 (100)	

* Chi-square test *p* < 0.05.

**Table 4:** Clinical characteristics of patients over the three study visits. Data is presented as median (IQR). Indicates statistically significant differences in the observed parameters (Chi-square test, *p* < 0.05).

	Median (IQR) Total			*p* *
	1st Visit	2nd Visit	3rd Visit	
**Creatinine (µmol/L)**	749 (617–905.5)	738 (602.8–927.3)	736.5 (635.3–951.5)	0.57
Urea (mmol/L)	21 (18.4–23.6)	20.9 (18.3–24.9)	22.65 (18.6–25)	0.26
Total protein (g/L)	66 (64–70)	66 (64–70.5)	67 (64–71)	0.29
Albumin (g/L)	38.25 (35.7–40)	38.6 (36.2–40.9)	38.4 (35.9–40.9)	0.37
Prealbumin (g/L)	0.5 (0.4–0.6)	0.5 (0.5–0.7)	0.5 (0.4–0.7)	**0.02**
Hemoglobin (g/L)	106 (99–116)	110 (104–117.25)	114 (103–120)	**0.04**
Leucocytes (×10^9^/L)	5.9 (4.8–7.4)	5.8 (4.88–6.93)	5.7 (4.7–6.4)	0.32
Thrombocytes (×10^9^/L)	171 (137.5–212)	164.5 (130.75–200.25)	166 (131–209.3)	0.19
Iron (µmol/L)	11 (8–14)	11 (8–14)	11.5 (9–14.3)	0.71
TIBC	39 (35.5–44)	38 (34–42.25)	38.5 (34.8–42)	0.19
Ferritin (µg/L)	391.1 (255.9–513.6)	397.5 (249.53–523.18)	350.75 (206.1–487.5)	0.17
EPO dose (IU per month)	24,000 (16,000–48,000)	32,000 (20,000–48,000)	32,000 (23,000–48,000)	0.87
Iron therapy (mg per month)	100 (0–200)	125 (0–125)	125 (31.3–125)	0.18
Calcium (mmol/L)	2.2 (2.12–2.3)	2.23 (2.1–2.3)	2.21 (2.1–2.3)	0.23
Phosphorus (mmol/L)	1.4 (1.14–1.71)	1.46 (1.2–1.8)	1.57 (1.3–1.8)	0.13
Potassium (mmol/L)	5 (4.45–5.4)	5.05 (4.4–5.5)	5.3 (4.7–5.9)	**<0.001**
Glucose (mmol/L)	5.7 (4.8–7.7)	5.9 (4.6–7.7)	5 (4.3–7.4)	**0.02**
CRP (mg/L)	4.75 (1.2–10.6)	3.5 (1.3–8.5)	4.1 (2.1–8)	0.26
Cholesterol (mmol/L)	4.05 (3.43–4.88)	3.9 (3.3–4.8)	3.9 (3.3–4.5)	0.21
Triglycerides (mmol/L)	1.57 (1.03–2.22)	1.45 (1–2.3)	1.29 (1–1.7)	0.64
HDL (mmol/L)	1.01 (0.82–1.27)	1.01 (0.8–1.3)	1 (0.8–1.2)	0.69
LDL (mmol/L)	2.23 (1.7–2.8)	2.01 (1.7–2.6)	2.16 (1.6–2.7)	0.32
BW (kg)	69.3 (56.3–81.7)	68.6 (56.5–81.5)	69.5 (57.5–79.4)	0.28
BMI (kg/m^2^)	24.5 (21.1–27.9)	24.3 (21.2–27.7)	25.4 (21.2–28.2)	0.50
MIS	8 (6–10)	8 (5–12)	6 (4.5–8.5)	**0.003**
OH (L)	2.4 (1.6–3.4)	2.4 (1.3–3.8)	1.9 (0.8–3)	**0.01**
LTI (kg/m^2^)	12.4 (10.2–14.3)	11.1 (9.8–13.6)	11.8 (9.8–14.1)	**0.009**
FTI (kg/m^2^)	11.3 (8.5–15.1)	12.05 (9.4–15.8)	11.9 (8.8–15.9)	0.08
NC (cm)	38 (35–41)	40 (35–41)	39 (35–42)	**0.03**
MUAC (cm)	27 (24–30)	28 (25–31.3)	29 (26–32)	**<0.001**
WC (cm)	93 (83–104.5)	95 (85–105)	98 (85–106)	**0.02**
HC (cm)	100 (93–107)	101 (95.5–107)	100.5 (95.9–109.3)	0.16
SST (mm)	14 (10–21)	13 (9–18.5)	15 (10–18.5)	**0.01**
TST (mm)	14 (10–20)	14 (8.5–21)	15 (9.5–20)	0.58

* Chi-square test, *p* < 0.05.

## Data Availability

The dataset generated and analyzed during this study contains individual patient-level clinical information. Due to privacy and ethical restrictions, these data cannot be made publicly available. De-identified data may be provided upon reasonable request to the corresponding author and with approval from the institutional ethics committee.

## Supplementary Materials

The following supporting information can be downloaded at: https://www.mdpi.com/article/10.3390/jcm14238337/s1, Table S1. The survivors-only longitudinal analysis, Table S2. Predictors of a mortality (multivariate regression analysis); *p* < 0.05 is considered statistically significant.

## Abbreviations

The following abbreviations are used in this manuscript:

CKD	Chronic kidney disease
MIA	Malnutrition–Inflammation–Atherosclerosis syndrome
PEW	Protein-energy wasting
ONS	Oral nutritional supplements
HD	Hemodialysis
MMAS-4	4-item Morisky Medication Adherence Scale
BDI	21-item Beck Depression Inventory
TIBC	Total iron-binding capacity
CRP	C-reactive protein
NC	Neck circumference
MUAC	Mid-upper arm circumference
WC	Waist circumference
HC	Hip circumference
TST	Triceps skinfold thickness
SST	Scapular skinfold thickness
BW	Body weight
BMI	Body mass index
LTI	Lean tissue index
FTI	Fat tissue index
OH	Overhydration
BCM	Body Composition Monitor
MIS	Malnutrition–Inflammation Score
ESRD	End-stage renal disease
EPO	Erythropoietin
