# Supplementary material for: Oral Nutritional Supplement Adherence and Nutritional Outcomes in Hemodialysis Patients—A Prospective Study
Source: J Clin Med. 2025 Nov 24;14(23):8337. doi: 10.3390/jcm14238337 (PMC12693438; doi:10.3390/jcm14238337)
Supplement: Supplementary file 1 [file jcm-14-08337-s001.zip › jcm-3931526-supplementary.pdf]

Oral Nutritional Supplement Adherence and Nutritional Outcomes in Hemodialysis Patients—A Prospective Study

Table S1. The survivors-only longitudinal analysis

	Median (IQR) total			p*
	1st visit	2nd visit	3rd visit	
Creatinine (µmol/L)	753 (628-933)	745 (620-936)	740 (641-955)	0.47
Urea (mmol/L)	20.8 (18.3-23.6)	21.2 (18.7-25.2)	22.7 (18.7-25)	0.23
Albumin (g/L)	38.9 (36.5-40.1)	38.8 (36.6-41.1)	38.5 (35.9-40.9)	0.34
Prealbumin (g/L)	0.5 (0.4-0.6)	0.6 (0.5-0.7)	0.5 (0.4-0.7)	**0.031**
Hemoglobin (g/L)	106 (98-115)	110 (104-118)	114 (103-120)	**0.032**
Leucocytes (x10⁹/L)	5.9 (4.8-7.4)	5.7 (4.8-6.9)	5.7 (4.8-6.4)	0.31
Thrombocytes (x10⁹/L)	176 (135-217)	165 (131-199)	167 (133-210)	0.232
Iron (µmol/L)	10.5 (8-14)	12 (8.5-15)	12 (9-14.5)	0.59
TIBC	39 (36-43)	38 (34-42)	38 (35-42)	0.19
Ferritin (µg/L)	388 (256-492)	402 (250-523)	355 (215-488)	0.21
Calcium (mmol/L)	2.19 (2.1-2.29)	2.23 (2.15-2.31)	2.21 (2.13 – 2.31)	0.27
Phosphorus (mmol/L)	1.42 (1.14-1.72)	1.5 (1.19-1.82)	1.57 (1.33 - 1.81)	0.09
Potassium (mmol/L)	5 (4.5-5.3)	5 (4.4-5.5)	5.3 (4.8-5.9)	**0.0001**
Glucose (mmol/L)	5.7 (4.8 – 7.4)	5.9 (4.7 – 7.7)	5 (4.3 – 7.4)	**0.013**
CRP (mg/L)	4.1 (1.15 – 4.1)	4 (2.1 – 7.9)	4 (2.1-7.9)	0.27
Cholesterol (mmol/L)	4 (3.4 – 4.8)	4 (3.3 – 4.9)	3.9 (3.3 – 4.5)	0.19
Triglycerides (mmol/L)	1.57 (1-2.22)	1.5 (1.02-2.27)	1.29 (0.97-1.76)	0.59
HDL (mmol/L)	0.98 (0.81-1.26)	0.97 (0.84-1.22)	0.99 (0.82-1.24)	0.74
LDL (mmol/L)	2.2 (1.73-2.72)	2.07 (1.74-2.67)	2.16 (1.63-2.76)	0.25
BW (kg)	71 (57-82)	70 (57-82)	70 (58-79)	0.27
BMI (kg/m²)	24.7 (21.3-28)	24.7 (21.4-27.9)	25.4 (21.2-28.2)	0.49
MIS	7 (5-9)	8 (5-11)	6 (4.5-8.5)	0.003
OH (L)	2.4 (1.5-3.4)	2.4 (1.2-3.8)	1.9 (0.8-3)	**0.011**
LTI (kg/m²)	12.5 (10.2-14.5)	11.4 (9.8-13.6)	11.8 (9.8-14.1)	**0.01**
FTI (kg/m²)	11.3 (8.6-15.1)	12.1 (9.1-15.7)	11.9 (8.8-15.9)	0.08
NC (cm)	38 (35-41.3)	40 (35-41.3)	40 (35-41.3)	**0.035**
MUAC (cm)	27.5 (25-30)	28 (26-31.6)	29 (26-32)	**0.001**
WC (cm)	92.5 (82-104.3)	95.5 (85-105)	98 (85-106)	**0.017**
HC (cm)	100 (93.8-107)	101.5 (97-107.3)	100.5 (95.9-109.3)	0.17
SST (mm)	14.8 (10-21)	14 (9.8-19.1)	15 (10-18.5)	**0.01**

Table S2. Predictors of a mortality (multivariate regression analysis); $p < 0.05$ is considered statistically significant.

Predictors	β	St. error	Wald	P	OR	95% CI
**1st visit**						
BW (kg)	-0.03	0.02	1.89	0.17	0.97	0.94-1.01
MIS	0.27	0.09	8.37	**0.004**	1.3	1.09-1.58
Albumin (g/L)	-0.28	0.09	8.86	**0.003**	0.76	0.63-0.91
Prealbumin (g/L)	-1.96	2.06	0.91	0.34	0.14	0.003-7.89
Cholesterol (mmol/L)	0.23	0.25	0.86	0.35	1.26	0.77-2.05
Triglycerides (mmol/L)	0.01	0.31	0.002	0.97	1.01	0.55-1.86
HDL (mmol/L)	0.24	0.58	0.17	0.68	1.27	0.41-3.93
LDL (mmol/L)	0.24	0.29	0.67	0.41	1.27	0.71-2.28
OH (L)	0.28	0.19	2.33	0.13	1.33	0.92-1.92
LTI (kg/m^2)	-0.14	0.11	1.57	0.21	0.87	0.70-1.08
FTI (kg/m^2)	-0.02	0.06	0.15	0.70	0.97	0.86-1.10
NC (cm)	0.03	0.06	0.27	0.60	1.03	0.91-1.17
MUAC (cm)	-0.19	0.08	5.32	**0.02**	0.83	0.70-0.97
WC (cm)	0.01	0.02	0.12	0.73	1.01	0.97-1.04
HC (cm)	-0.03	0.03	1.001	0.32	0.97	0.92-1.03
SST (mm)	-0.07	0.04	2.33	0.13	0.93	0.86-1.02
TST (mm)	-4.72	2.90	2.64	0.10	0.009	0-2.64
**2nd visit**						
BW (kg)	-0.03	0.02	1.7	0.19	0.97	0.92-1.02
MIS	0.14	0.08	2.93	0.08	1.16	0.97-1.37
Albumin (g/L)	-0.20	0.08	5.55	**0.02**	0.82	0.69-0.97
Prealbumin (g/L)	-2.57	2.46	1.09	0.39	0.08	0.001-9.5
Cholesterol (mmol/L)	-0.49	0.39	1.59	0.21	0.61	0.29-1.31
Triglycerides (mmol/L)	-0.85	0.61	1.93	0.16	0.43	0.13-1.42
HDL (mmol/L)	1.55	0.77	4.07	**0.04**	4.71	1.05-21.2
LDL (mmol/L)	-0.86	0.54	2.52	0.11	0.42	0.15-1.22
OH (L)	0.28	0.22	1.62	0.20	1.33	0.86-2.05
LTI (kg/m^2)	-0.21	0.16	1.67	0.20	0.81	0.59-1.12
FTI (kg/m^2)	0.01	0.07	0.04	0.84	1.01	0.88-1.17
ON (cm)	-0.06	0.09	0.34	0.56	0.94	0.78-1.15
MUAC (cm)	-0.25	0.12	4.11	**0.04**	0.78	0.61-0.99
WC (cm)	-0.007	0.03	0.08	0.78	0.99	0.94-1.05
HC (cm)	-0.03	0.03	1.04	0.61	0.97	0.92-1.03
SST (mm)	-0.16	0.09	3.23	0.07	0.85	0.72-1.01
TST (mm)	-0.34	0.14	6.24	**0.01**	0.71	0.54-0.92
